# Stress Reaction in Outer Segments of Photoreceptors after Blue Light Irradiation

**DOI:** 10.1371/journal.pone.0071570

**Published:** 2013-09-11

**Authors:** Cora Roehlecke, Ulrike Schumann, Marius Ader, Coy Brunssen, Silvia Bramke, Henning Morawietz, Richard H. W. Funk

**Affiliations:** 1 Institute of Anatomy, Technische Universität (TU) Dresden, Dresden, Germany; 2 Center for Regenerative Therapies Dresden (CRTD) DFG – Cluster of Excellence, Biotechnology Center, Dresden, Germany; 3 Division of Vascular Endothelium and Microcirculation, Department of Medicine III, TU Dresden, Dresden, Germany; University of Tasmania, Australia

## Abstract

The retina is prone to oxidative stress from many factors which are also involved in the pathogenesis of degenerative diseases. In this study, we used the application of blue light as a physiological stress factor. The aim of this study was to identify the major source of intracellular ROS that mediates blue light-induced detrimental effects on cells which may lead to cytotoxicity. We hypothesized that outer segments are the major source of blue light induced ROS generation. In photoreceptors, nicotinamide adenine dinucleotide phosphate (NADPH) oxidase (Nox) enzymes and the recently found respiratory chain complexes may represent a major source for reactive oxygen species (ROS), beside mitochondria and chromophores. Therefore, we investigated this hypothesis and analysed the exact localization of the ROS source in photoreceptors in an organotypic culture system for mouse retinas.

Whole eyeball cultures were irradiated with visible blue light (405 nm) with an output power of 1 mW/cm^2^. Blue light impingement lead to an increase of ROS production (detected by H_2_DCFDA in live retinal explants), which was particularly strong in the photoreceptor outer segments. Nox-2 and Nox-4 proteins are sources of ROS in blue light irradiated photoreceptors; the Nox inhibitor apocynin decreased ROS stimulated by blue light. Concomitantly, enzyme SOD-1, a member of the antioxidant defense system, indicator molecules of protein oxidation (CML) and lipid oxidation (MDA and 4-HNE) were also increased in the outer segments.

Interestingly, outer segments showed a mitochondrial-like membrane potential which was demonstrated using two dyes (JC-1 and TMRE) normally exclusively associated with mitochondria. As in mitochondria, these dyes indicated a decrease of the membrane potential in hypoxic states or cell stress situations.

The present study demonstrates that ROS generation and oxidative stress occurs directly in the outer segments of photoreceptors after blue light irradiation.

## Introduction

Oxidative stress is considered to be a major factor in the pathogenesis of degenerative diseases of the retina including age-related macular degeneration (AMD) [1]. Furthermore, the antioxidant capacity in the retina (e.g. via macular molecules like lutein and zeaxanthin) is reduced in AMD patients [2]. Indeed, compared to other tissues, the retina is particularly prone to the generation of reactive oxygen species (ROS) due to the very high oxygen levels in the choroid, the extraordinary high metabolic rates and exposure to light, especially light of shorter wavelengths [1], [3], [4], [5], [6], [7]. Furthermore, lipids of outer segment membranes of photoreceptors (with a very high amount of polyunsaturated fatty acids, PUFA) can be oxidized by radicals produced during these processes.

Because of the extremely high oxygen gradient from the choroid to the inner segment of the photoreceptors [8], it has been suggested that the oxygen consuming mitochondria in inner segments play the primary role in oxidative stress reactions of the outer retina [9], [10]. As discussed by these authors, mitochondria represent a major source of endogenous ROS in the photoreceptors and the underlying RPE. Indeed, mitochondria are particularly sensitive to oxidative stress due to the handling of electrons in the respiratory chain [11]. In addition, after blue light exposure, more electrons deviate from the respiratory chain in the mitochondria, resulting in further damage: in fact, inhibiting the mitochondrial transport chain in RPE cells or addition of mitochondria-specific antioxidants blocks ROS formation and cell death [4]. Furthermore, chromophores in general, especially the cytochromes can be sources of ROS [2], [4].

The NADPH oxidase (Nox) family of enzymes has recently been recognized as an generator of ROS in photoreceptors after damage by serum deprivation [12] or cone cells in a model of retinitis pigmentosa [13]. We have previously demonstrated that blue light irradiation resulted in increased superoxide anion production [7]. It is undetermined whether Nox proteins contribute to the generation of ROS by blue light irradiation.

The new findings that enzymes of the respiratory chain are also located in the membranes of the outer segments give the topic of ROS generation in photoreceptors an exciting new perspective [14], [15], [16]. These studies demonstrated that the activity of respiratory chain complexes in outer segment fractions was comparable to that found in retinal mitochondria-enriched fractions. They showed that in isolated outer segments a proton potential difference exists across the disk membranes, similarly formed as double membranes as the double membranes of the mitochondria. This implies that the outer photoreceptor segment respiratory complexes might also be able to generate ROS.

The aim of the present study was to investigate ROS in outer segments of photoreceptors after blue light irradiation. We hypothesized that outer segments are the major source of blue light induced ROS generation. ROS derived from Nox proteins may be essential for triggering blue light damage. In addition to Nox proteins, we are looking for evidence of involvement of extra-mitochondrial respiratory complexes in outer segments in blue light damage. For this purpose it is necessary to investigate ROS production and mitochondrial membrane potential in real time in the retina, particularly in the outer segments of photoreceptors.

Using an organotypic culture system for mouse retinas, we recently demonstrated that oxidative damage is a major contributing factor to photoreceptor cell death after blue light exposure [7]. The advantage of using this culture system is that the photoreceptors and their outer segments are in good order and faultless allowing for a detailed in situ analysis and examination of ROS production in real time in live retinal tissue. In contrast, methods relying on isolated photoreceptors are unsuitable because the very few photoreceptor cell lines that are available do not produce outer segments. To obtain isolated photoreceptors with outer segments, primary cells must be isolated from intact retinas, but outer segments are very fragile and are prone to shearing off during the photoreceptor cell isolation process. In addition, isolation of outer segments very likely causes cumulative damage to the outer segments that would compound effects provoked by blue light irradiation, thus aggravating the analysis of blue light induced damages. Using the same model, we found that the outer segments (with their newly found respiratory complex activity) produce massive amounts of ROS under blue light stress – more than the mitochondria of the inner segment. Another important outcome of our study is the corroboration of the above mentioned mitochondria-like activity in the outer segments via special dyes which normally show exclusively the functional state of mitochondrial membranes.

## Methods and Materials

### Organ culture

The organotypic model of photoreceptors is well established and has already been characterized in detail [7]. On postnatal day 24±4 days (shortly after weaning), C57BL/6 mice of either sex were sacrificed by cervical dislocation. Their eyes were immediately enucleated and transferred into phosphate buffered saline (PBS). The eyeballs were punctured with a needle (BD Microlance 3, 27G×0.5 Inch) to create a small hole which enabled fluid exchange and were transferred into an optimized cell culture medium. The eyeballs were cultivated in almost their original form in medium (DMEM/F12 GIBCO (cell culture medium)+10% fetal calf serum (FCS)+2% B-27 supplement+1% penicillin-streptomycin+2 mM glutamine) in a 6-well culture plate at 37°C with a CO_2_ level of about 5% in a cell culture incubator for different lengths of time. Where indicated, eyeballs were cultivated in medium with 4 mM apocynin (Abcam, Cambridge, UK).

### Ethics Statement

All animal experiments were approved by the ethics committee of the TU Dresden and the license for removal of organs was provided by the Landesdirektion Dresden (Az.: 24D-9168.24-1/2007-27).

### Irradiation with blue light

Illumination was produced by a LED-based system (# LZ1-00UA05 BIN U8; LedEngin, Santa Clara, USA) that was constructed in our lab [7]. It generated short wavelength blue light (peak at 405 nm) with an output power of 1 mW/cm^2^. The eyes were positioned in cut cell culture inserts (transparent; BD, Heidelberg, Germany) so that their corneas faced the blue light diodes (1 per well). Non-irradiated eyes were used as the controls.

### Measurement of intracellular reactive oxygen species (ROS) production

For evaluation of ROS production in the photoreceptors we used a dye for live staining – 5-(and-6)-chloromethyl-2′,7′-dichlorodihydrofluorescein diacetate, acetyl ester (CM-H_2_DCFDA; Molecular Probes®-Invitrogen, Darmstadt, Germany). First, the retinas were dissected after 0.5 and 1 h blue light exposure, respectively. Next, the retinal explants were loaded with 25 µM CM-H_2_DCFDA (in PBS) for 10 min at 37°C in a cell culture incubator. CM-H_2_DCFDA is non-fluorescent until the acetate groups are removed by intracellular esterases and oxidation occurs within the cell. Then it yields green fluorescence (excitation ∼492–495 nm/emission 517–527 nm). CM-H_2_DCFDA detects ROS production in form of hydrogen peroxide (H_2_O_2_), peroxynitrite anions (ONOO^−^), hydroxyl radicals (**^.^**OH) or peroxide radicals (ROO**^.^**). After the staining, the samples were rinsed once in PBS, then transferred to 4% paraformaldehyde (PFA) and immediately fixed for at least one to two hours. Then, the retinal explants were embedded in 4% agarose and cut in 40 µm vertical sections using a vibratome (VT1200 S; Leica Microsystems, Wetzlar, Germany) for a suitable determination of ROS in the different layers. The sections were mounted on glass slides and without delay the slides were analyzed using a LSM 510 confocal laser scanning microscope (Carl Zeiss, Jena, Germany) and IX-81 inverted microscope (Olympus, Jena, Germany), respectively. The time frame between vital staining of the tissue and analyzing the images was up to six hours. Images were obtained using an Apo-40× objective. Same acquisition settings were used throughout all experiments for each microscope to allow direct comparison of retinal explants treated with or without blue light and with or without apocynin, respectively. Digital images were processed using ImageJ free software (Rasband, W.S., ImageJ; U.S. NIH, Bethesda, USA). Only cropping of the images was performed – there was no adjustment to the brightness. The mean fluorescence intensity ratio of outer segments and inner segments was determined in 10 different regions of interest (same size) from one retinal section per time point (each of them as representative of 3 experiments). The regions were distributed equally over each full respective layer. The ratio between irradiated outer or inner segments (numerator) and non-irradiated, time-matched control inner segments (denominator) was calculated to determine increases in the general ROS production in specific treatment groups.

### Measurement of mitochondrial membrane potential

Mitochondrial membrane potential (MMP) was assessed by measuring the potential-dependent accumulation of 5,5′,6,6′-tetrachloro-1,1′,3,3′ tetraethylbenzimidazolylcarbocyanine iodide (JC-1) [17], [18], [19] or tetramethylrhodamine, ethyl ester (TMRE) [20] which also apparently also the membranes in the outer segments of the photoreceptors. Retinas were freshly prepared as whole mounts from animals after decapitation and enucleation. Immediately afterwards, they were incubated with either 10 µg/ml JC-1 or 20 nM TMRE. The procedure from killing to obtaining the first images took ca. 2 min. Additionally the organotypic cultures were cultivated and irradiated for 6 h and 12 h. The retinal whole mounts were prepared and stained for 10 min as mentioned before.

### Immunohistochemistry

After the cultivation periods, each eyeball was fixed for 30 min in 4% paraformaldehyde (PFA) at room temperature. Then the eyeball was cut in half through the equator to permit removal of the anterior segment and vitreous body and lens. The remaining eyecup was fixed overnight at 4°C in 4% PFA. After fixation the eyecups were cut in half or quarters and put in embedding cassettes. Before embedding the samples were pretreated at room temperature for 4 times 20 min in 1× PBS, then 1 h 70% (v/v) ethanol (EtOH), 1 h 96% (v/v) EtOH, 1 h EtOH 100% (v/v) and 30 min in xylene. It followed the embedding in paraffin at 65°C for 2 h. The retinas were positioned in a way to enable subsequent sagittal sections and allowed to cool.

Sagittal sections of the eyecups were cut at 7 µm with a microtome (RM2065; Leica Microsystems, Wetzlar, Germany). The sections were flattened out in a hot water bath (ca. 40–45°C) and then mounted on slides, which were pre-silanized to enhance tissue adherence. The slides were dried overnight at 37°C. For storing they were cooled down to room temperature. The slides were deparaffinized in xylene for 3×5 min and hydrated for 3×2 min in 100% (v/v) EtOH, 2×2 min in 96% (v/v) EtOH, 2 min in 70% (v/v) EtOH, 2 min in 40% (v/v) EtOH. Then they were rinsed 2 times in distilled H_2_O.

If heat induced antigen retrieval was recommended, then the deparaffinized slides were placed in a cuvette in citrate buffer containing citric acid monohyrate and tri-sodium citrate dehydrate in H_2_O, and cooked for 2 times 5 min in the microwave at 800 W. After cooling for 15 min, the slides were rinsed in distilled H_2_O and then 2 times for 5 min in PBS buffer. The sections were blocked in blocking solution (PBS+5% normal goat serum+0.3% Triton X 100+1% bovine serum albumine (BSA)) for 1 h at room temperature and incubated with the primary antibody (diluted in PBS) overnight in a humid chamber at 4°C. The next day the slides were washed 2 times for 5 min in PBS and afterwards incubated with the fluorescence-linked secondary antibody (diluted in PBS) for 30 min at 37°C. After that the slides were washed 2 times for 5 min in PBS. The sections were stained for 10 min with DAPI (Sigma-Aldrich, 1∶50 in PBS) to allow a better identification of the retinal layers, shortly rinsed in PBS, and a coverslip was mounted on the section with DABCO mounting medium. If necessary the coverslip was fixed with nail polish to avoid further movement.

Fluorescence images were obtained using an Axio Imager Z1 microscope (Carl Zeiss). The same acquisition settings were used throughout all experiments to allow direct comparison of retinal explants treated with or without blue light. Digital pictures were acquired, stored, and visualized with AxioVision 4.7 Software (Carl Zeiss).

Antibodies used for this method: polyclonal rabbit anti-Nox-4 (ab60940; Abcam, Cambridge, UK; dilution 1∶200), polyclonal rabbit anti-gp91-phox ( = anti-Nox-2; 07-024; Millipore, Schwalbach, Germany; dilution 1∶50), polyclonal rabbit anti-MDA (PAB14723; Abnova, Heidelberg, Germany; dilution 1∶100), monoclonal mouse anti-4-HNE (MAB6115; Abnova; dilution 1∶20), polyclonal rabbit anti-CML (gift from Prof. Schleicher; dilution 1∶1000), monoclonal mouse anti-SOD-1 (clone 30F11; Novocastra Laboratories Ltd., Newcastle upon Tyne, UK; dilution 1∶500), secondary antibodies (Dianova; Germany; dilution 1∶100).

### Western blot analysis

After the cultivation periods, the retinas were removed and for each sample two to three retinas were put directly in 80 µl of lysis buffer including (60 mM Tris-HCl, 1% (m/v) SDS, 1 mM Na_3_VO_4_ in distilled H_2_O+protease inhibitor Complete; Roche Diagnostics, Mannheim, Germany) in a 2 ml tube. The samples were homogenized using an ultrasonic processor (Hielscher Ultrasonics, Teltow, Germany), followed by an incubation on ice for 30 min. Next, the samples were centrifuged at 12000 g for 5 min at 4°C. The supernatant including the proteins of interest was kept in a new 1.5 ml tube and the pellet was discarded. The proteins were directly frozen at −80°C at this point or were subjected to a BCA-assay for determining the protein concentration in each sample.

The preparation of outer segments was done according to previous protocols [21], [22]. To better protect the tissue, protease inhibitor (Complete; Roche Diagnostics) was added to the solution. We pooled 8–10 mouse retinas for each sample to obtain enough outer segment material.

Total protein of the lysate supernatant was determined using BCA Protein Assay Kit (Thermo Scientific, Rockford, USA) and 10 µg of total protein of each sample redissolved in 6× SDS sample buffer (300 nM Tris-HCl, pH 6.8; 30% (w/v) glycerol; 10% (w/v) SDS; 0.1% bromophenol blue; 100 mM DTT). After boiling the samples for 5 min at 95°C they were loaded on a 10% SDS-polyacrylamide gel. The separated proteins were transferred to a 0.45 µm PVDF-membrane (Immobilon-P™; Millipore, Schwalbach, Germany). After blocking the PVDF-membrane in TBS-T (137 mM NaCl, 2.7 mM KCl, 20 mM Tris–HCl, 0.2% Tween 20; pH 7.4) containing 5% non-fat dry milk, it was incubated with polyclonal rabbit anti-Nox-4 (ab60940; Abcam, Cambridge, UK; dilution 1∶500), polyclonal rabbit anti-gp91-phox ( = anti-Nox-2; 07-024; Millipore, Schwalbach, Germany; dilution 1∶500), polyclonal rabbit anti-MDA (PAB14723; Abnova, Heidelberg, Germany; dilution 1∶500), or rabbit polyclonal anti-β actin (NB100-78420; Novus Biologicals, Littleton, CO, USA; dilution 1∶1000), for 2 h at RT or overnight at 4°C. The membrane was washed three times for 10 min then incubated with secondary HRP conjugated antibodies (ECL anti-rabbit IgG or ECL anti-mouse IgG; GE Healthcare, Little Chalfont, UK; dilution 1∶5000 or 1∶2000, respectively) for 1 h at RT followed washing again (three times for 10 min). Chemiluminescent signal was generated using Immobilon Western Chemiluminescent HRP Substrate (Millipore, Billerica, MA, USA) and detected with an Image Reader LAS-3000 (Fuji Photo Film, Tokyo, Japan). Protein quantification was performed with ImageJ free software (Rasband, W.S., ImageJ; U.S. NIH, Bethesda, USA). Each lane was quantified along with the corresponding loading control (β-actin).

### Real-Time-PCR

The isolation of RNA of whole retinas was carried out according to the manual of the RNeasy® Mini Kit (Qiagen, Hilden, Germany). The RNA concentration was measured with a NanoPhotometer (Implen, München, Germany).

Reverse transcription of mRNA into cDNA was performed using SuperScript II Reverse Transcriptase according to manufacturer's instructions (Life Technologies, Darmstadt, Germany). Equal amounts of total RNA (500 ng) were incubated for 3 min at 70°C and subsequently reverse transcribed into cDNA using random hexamer primers for 1 h at 42°C. Quantification was performed by real-time PCR with GoTaq qPCR Master Mix (Promega, Mannheim, Germany) as described previously [23]. Rpl32 was used as reference gene for cDNA content normalization. Amplification started with an initial denaturation step at 95°C for 2 min, followed by 45 cycles of denaturation at 95°C for 20 s, annealing for each gene at 60°C for 30 s, and extension at 72°C for 10 s. After final extension at 72°C for 2 min, melt-curve analysis was performed following every run to ensure a single amplified product in each reaction.

Following primers were used:

Nox-2 forward 5′-AGCTATGAGGTGGTGATGTTAGTGG-3′,

Nox-2 reverse 5-′CACAATATTTGTACCAGACAGACTTGAG-3′,

Nox-4 forward 5-′TGTTGGGCCTAGGATTGTGTT-3′,

Nox-4 reverse 5-′AGGGACCTTCTGTGATCCTCG-3′

Rpl32 forward 5′-GCGCTGCCTACGAGGTGGCTG-3′,

Rpl32 reverse 5′-CTGGCCCTTGAACCTTCTCCGC-3′.

Analysis of the raw data was performed with the iQ5 software (Bio-Rad, Munich, Germany). Evaluation of the data was done using a mathematical model of relative expression ratio in real-time PCR under constant reference gene expression [24].

### Statistical analysis

Data are presented as mean ± standard error of mean (SEM). One-way analysis of variance (ANOVA) was used throughout. When significance was achieved, it was followed by post hoc Bonferroni test. Statistical analysis was performed using GraphPad Prism 5.03 (GraphPad, SanDiego, CA, USA) and significance was accepted at *p<0.05. For qPCR we used t-test and paired t-test. We accomplished a minimum of three independent experiments.

## Results

### Increase of ROS production in outer retinal layers after blue light damage

**Figure 1:**
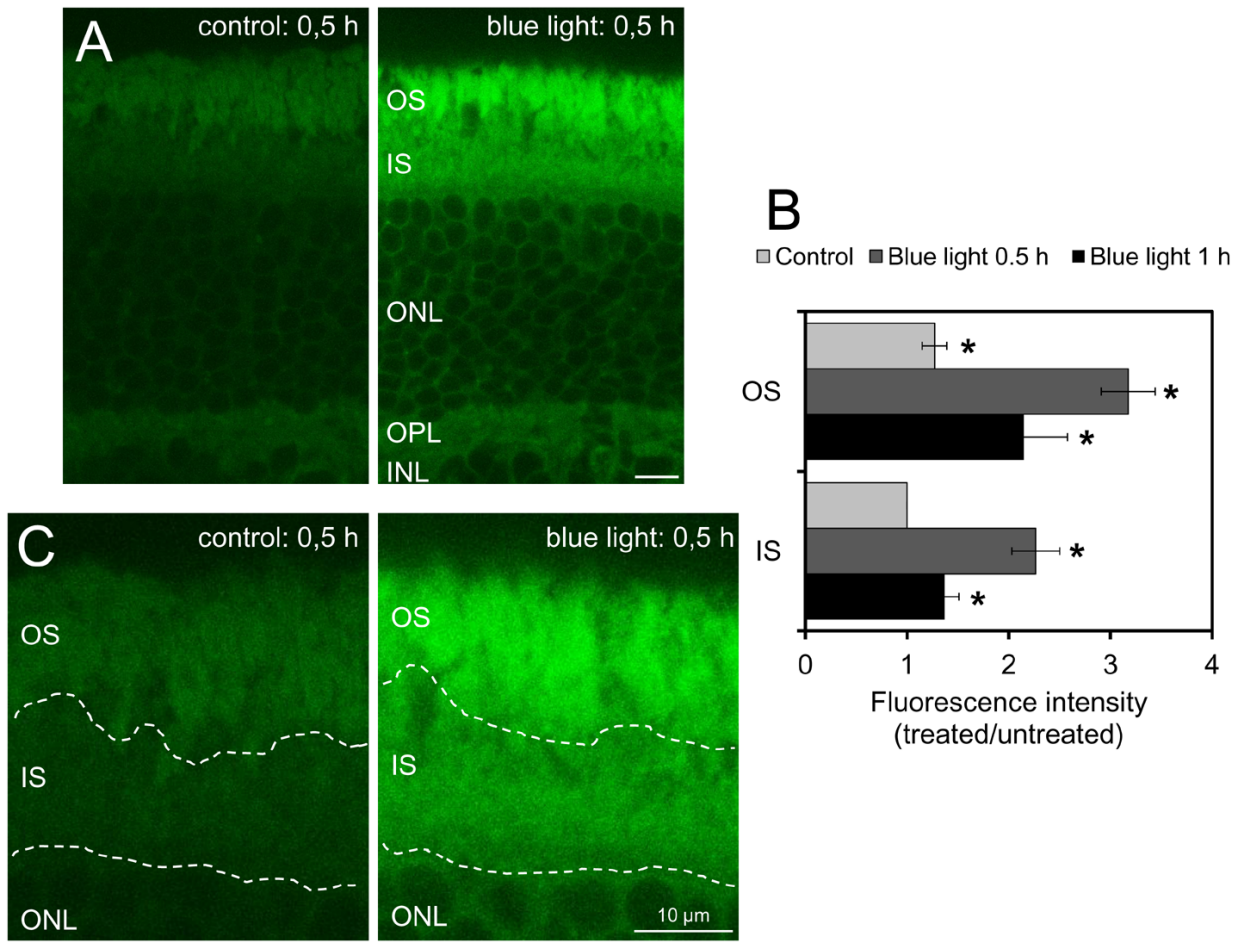
Reactive oxygen species (ROS) production is increased in outer retinal layers after blue light damage, particularly in outer segments. **A.** Confocal laser scanning microscopy images of 40 µm vibratome sections of retinas are presented. After 0.5 h of blue light exposure, irradiated explants and respective non-irradiated explants (controls) were loaded with 25 µM 5-(and-6)-chloromethyl-2′,7′-dichlorodihydrofluorescein diacetate, acetyl ester (CM-H_2_DCFDA; ROS indicator). Irradiated explants showed higher fluorescence intensity in OS and IS compared to time-matched controls (same microscope settings). Scale bar represents 10 µm; images are representative of 3 experiments **B.** Quantitative analysis of ROS production in outer and inner segments of retinal explants. Retinas were exposed to visible blue light for 0.5 h and 1 h. The graph displays the mean fluorescence intensity ratios of irradiated photoreceptor cell layers versus non-irradiated time-matched controls, the IS of the controls are normalized to 1 (determined by Image J software). Bars represent the mean ± standard error of mean (SEM) from n = 10 different equal regions of interest (ROIs; * shows significance compared to IS-control; *p<0.05 determined by ANOVA, post hoc Bonferroni test). **C:** Magnification of confocal laser scanning microscopy images of the outer retinal layers of figure 1 after 0.5 h of blue light exposure. The ROS production in the outer segments of the photoreceptors is increased more than in the inner segments. This is indicated by a more intense fluorescence of CM-H_2_DCFDA in the outer segments than in the inner segments after irradiation periods with blue light. **OS**: outer segments; **IS**: inner segments; **ONL**: outer nuclear layer; **OPL**: outer plexiform layer; **INL**: inner nuclear layer.

To evaluate the exact localization of ROS production in photoreceptors, we treated live retinal explants with blue light for 0.5 h or 1 h. Intracellular ROS production was measured by incubating the tissue with the ROS indicator CM-H_2_DCFDA, confirming an increased ROS generation in photoreceptors after blue light exposure (Figure 1A). A 0.5 h exposure to blue light stimulated the greatest amount of ROS formation, as evidenced by the oxidation of CM-H_2_DCFDA (Figure 1B). To quantify the level of ROS production in inner segments (ISs) and outer segments (OSs) the intensity of the fluorescence signal was analysed by Image J software. In both the IS and OS, ROS production was stimulated by blue light exposure. ROS production increased 2.3-fold in IS and 3.2-fold in OS after 0.5 h compared to the basic fluorescence in the control IS. Furthermore, there was 1.4-fold increase in IS and 2.1-fold increase in OS after 1 h. In the OS of the controls, more ROS was produced than in the IS (Figure 1B, 1C).

### Increase of stress-relevant proteins in outer segments after blue light damage

In prior studies we showed that blue light irradiation resulted in increased superoxide anion production [7]. Therefore, we evaluated the expression of NADPH oxidases (Nox) proteins, which produce superoxide anions. Nox enzymes are transmembrane carriers and because the membranes in the outer segments were damaged after blue light exposure [7], we hypothesized an influence of blue light on these proteins.

**Figure 2:**
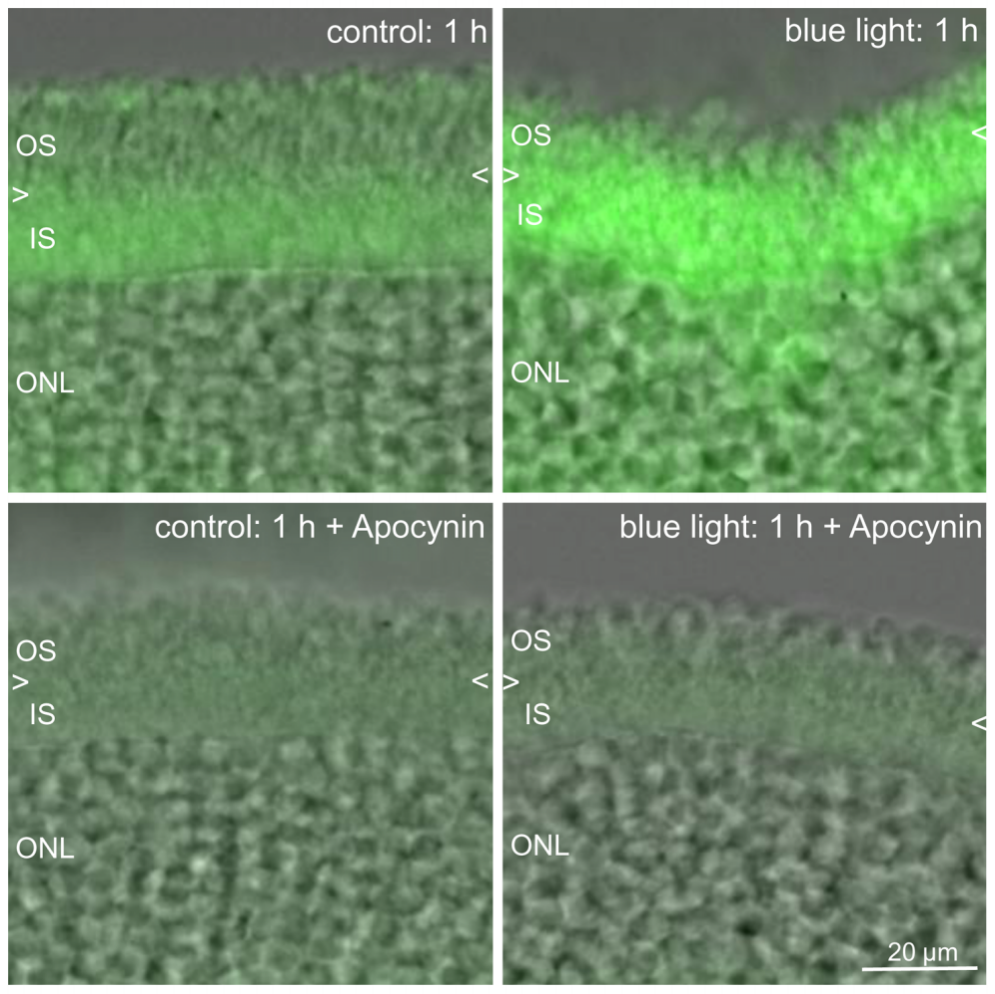
ROS production is reduced by the Nox inhibitor apocynin. Merged CM-H_2_DCFDA fluorescence and bright field microscopy images of 40 µm vibratome sections of retinas are presented. After 1 h of blue light exposure, irradiated explants and respective non-irradiated explants (controls) were loaded with 25 µM CM-H_2_DCFDA. In some cases, explants were pretreated with 4 mM apocynin during blue light exposure. The Nox inhibitor apocynin effectively reduced the levels of ROS production in the photoreceptors. The arrowheads mark the assumed border between IS and OS. The images are representative of 3 experiments. OS: outer segments; IS: inner segments; ONL: outer nuclear.

**Figure 3:**
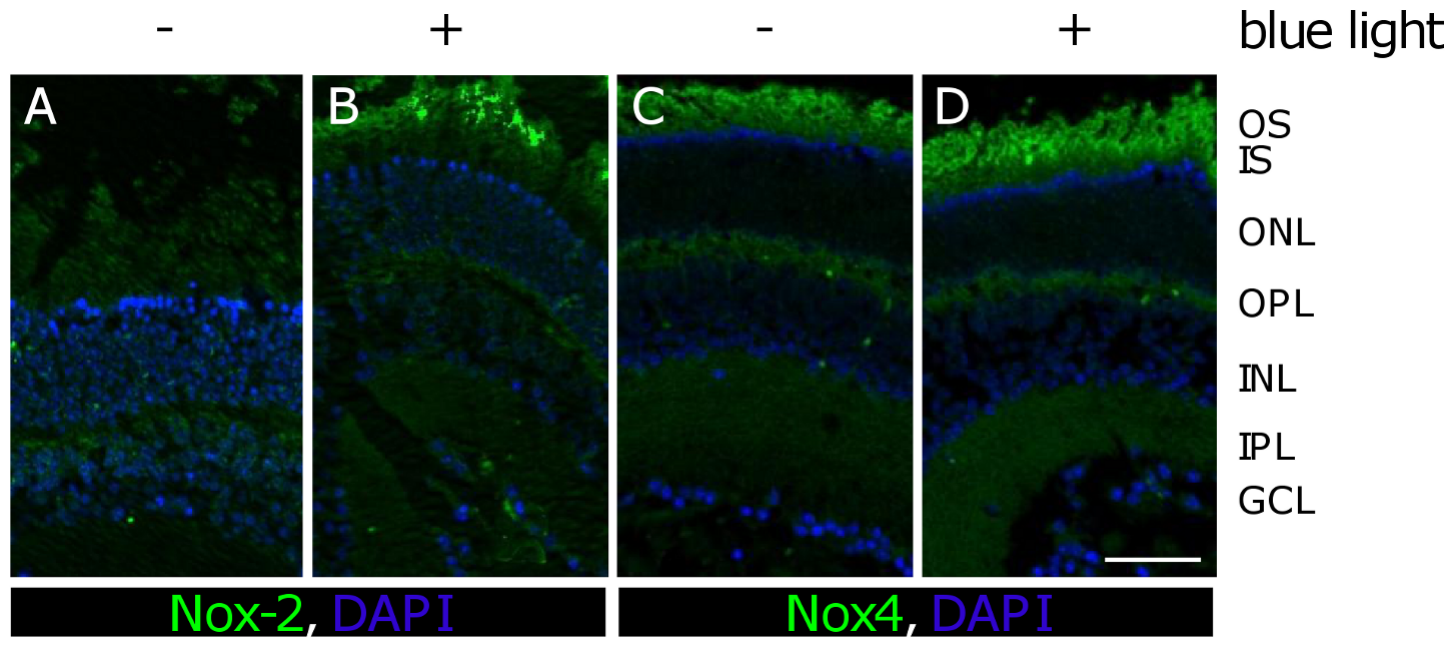
Immunofluorescence intensity of Nox-2 and Nox-4 proteins increased after 12 h of blue light exposure. **A, C,** Paraffin sections of retinas after 12 h of cultivation. **B, D,** Paraffin sections of retinas after 12 h of blue light exposure. Nox-2 was increased in the OS (**B**) while Nox-4 immunofluorescence intensity appeared slightly increased compared to the control (**C, D**). **A–D**, scale bar 50 µm; images are representative of n = 3 experiments **OS**: outer segments; **IS**: inner segments; **ONL**: outer nuclear layer; **OPL**: outer plexiform layer; **INL**: inner nuclear layer; **IPL**: inner plexiform layer; **GCL**: ganglion cell layer.

**Figure 4:**
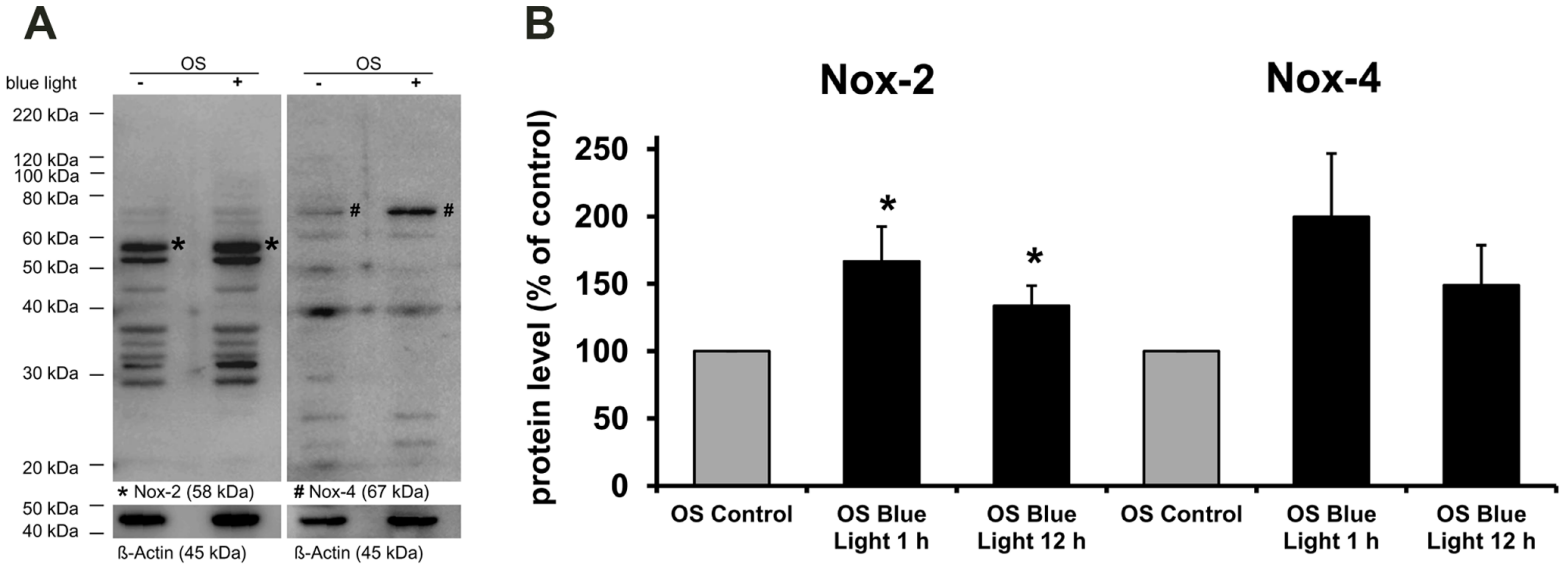
Effect of blue light on Nox-2 and Nox-4 protein expression. **A,**Western blot analysis showing increased Nox-2 and Nox-4 protein expression in OS following 1 h of blue light exposure (+) or in controls. The blots were first exposed to anti-Nox-2 or anti-Nox-4, respectively and then to anti-beta-Actin antibody as loading control. Images are representative of 5 experiments. **B,** Bar chart of densitometric analysis of Nox-2 and Nox-4 expression after 1 h and 12 h compared to control beta Actin. Bars represent the mean ± SEM from n = 5 experiments (* shows significance compared to control; *p<0.05 determined by ANOVA, post hoc Bonferroni test).

Blue light irradiation increased Nox proteins in the outer segments of photoreceptors. To further investigate if Nox proteins contributed to the generation of ROS, apocynin was used [12]. When retinal whole mounts were treated with 4 mM apocynin, ROS was substantially reduced (Figure 2). Also, we detected an increase of Nox-2 protein in the outer segments of photoreceptors that were exposed to blue light irradiation for 12 h compared to their time-matched controls (Figure 3). Nox-4 expression in the outer segments of the blue light damaged retina was slightly changed compared to the time-matched control after 12 h (Figure 3). The differences in the expression of Nox-2 were confirmed by Western Blot analysis (Figure 4). However, after 1 h irradiation, Nox-2 and Nox-4 proteins are increased compared to their time-matched controls (Fig. 4A and 4B).

**Figure 5:**
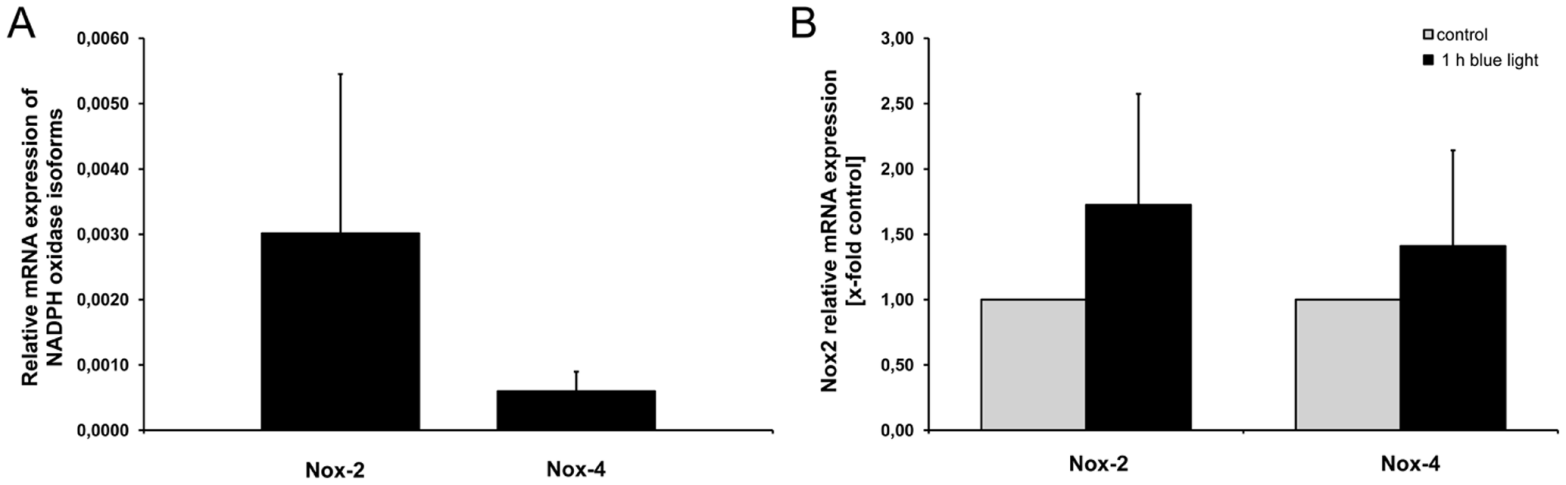
mRNA expression of NADPH oxidase isoforms in retinas. A, NADPH oxidase (Nox) isoforms Nox-2 and Nox-4 are expressed in the retina of untreated control mice. **B,** Nox-2 and Nox-4 mRNA expression after 1 h of blue light exposure of irradiated retinas compared to time-matched non-irradiated controls. The mRNA expression was quantified by real-time PCR. Rpl32 was used as reference gene. Data are shown as relative expressions ± SEM (A) or as x-fold of time-matched controls ± SEM (B). Statistics: t-test (A; p = 0.341) or paired t-test (B; Nox-2: p = 0.272; Nox-4: p = 0.239), n = 9.

Additionally to the immunohistochemical analysis of Nox-2 and Nox-4 we determined the mRNA expression of both Nox isoforms. Total RNA from retina homogenates was prepared and subjected to real-time PCR. Nox-2 and Nox-4 were the major Nox isoforms in murine retina (Figure 5A). Nox-2 showed the highest expression. In contrast, Nox isoforms Nox-1 and Nox-3 were below the level of detection (data not shown). Both Nox-2 (1.7-fold, Figure 5B) and Nox-4 (1.4-fold, Figure 5B) mRNA expression showed a trend to be increased after 1 h blue light irradiation compared to the time-matched control sample without reaching statistical significance (n = 9).

**Figure 6:**
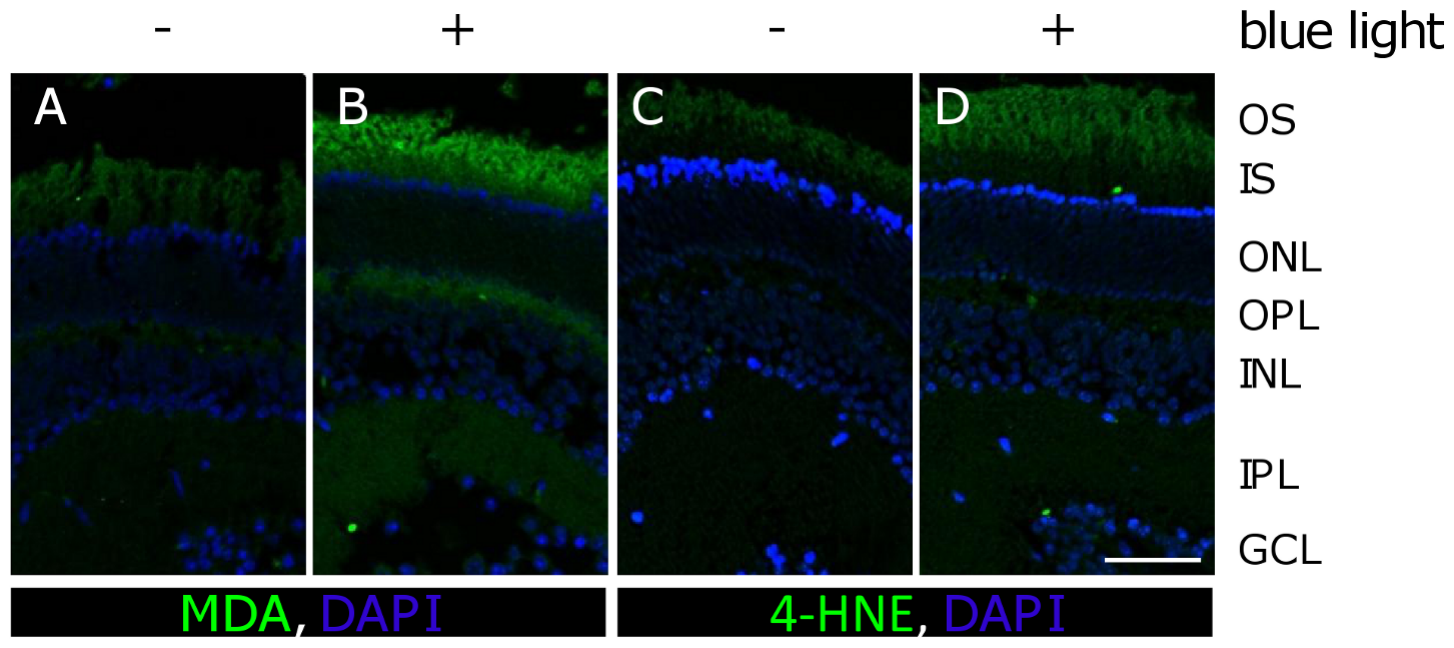
Expression of malondialdehyde (MDA) and 4-hydroxy-nonenal (4-HNE) increased after 12 h of blue light exposure in outer segments. **A, C,** Paraffin sections of retinas after 12 h of cultivation. **B, D,** Paraffin sections of retinas after 12 h of blue light exposure. Oxidative stress caused by blue light exposure led amongst others to lipid peroxidation and end-products like MDA and 4-HNE increased considerably in the OS (**B, D**). The respective time-matched controls showed only a weak autofluorescence in the OS (**A, C**). **A–D**, scale bar 50 µm; **OS**: outer segments; **IS**: inner segments; **ONL**: outer nuclear layer; **OPL**: outer plexiform layer; **INL**: inner nuclear layer; **IPL**: inner plexiform layer; **GCL**: ganglion cell layer.

**Figure 7:**
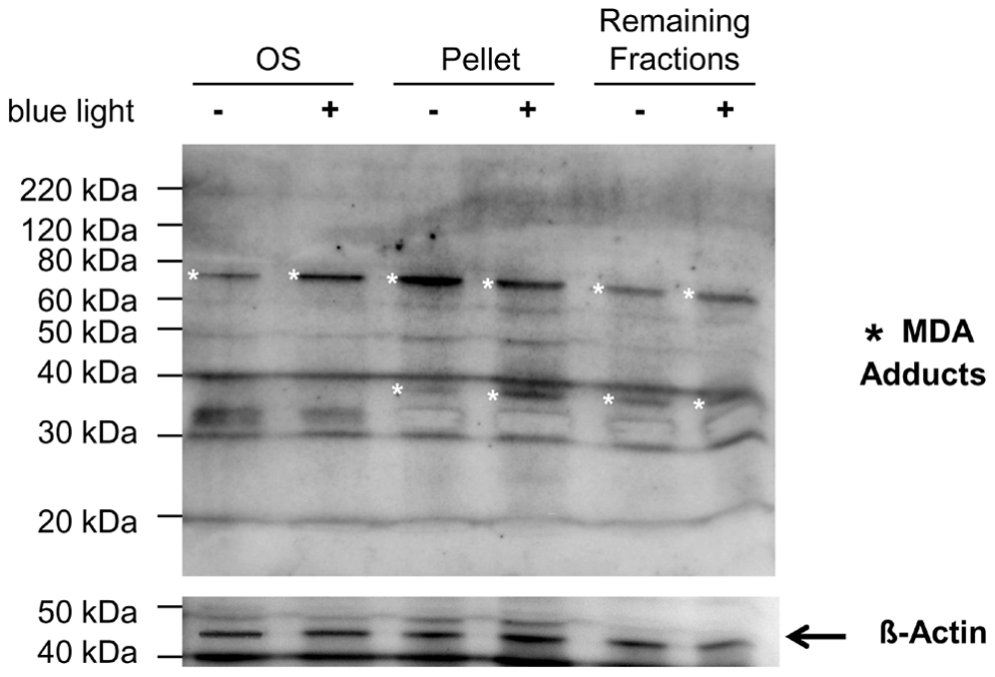
Effect of blue light on MDA adducts. Western blot analysis of MDA adducts after 12 h blue light exposure and in controls. The blot was first exposed to anti-MDA and then to anti-ß-Actin antibody as loading control. From the retina that was irradiated with blue light (+) or not (−), were different fractions loaded on a gel – isolated outer segments (OS), cytosolic proteins in the pellet and other proteins (remaining fractions). The expression of different MDA adducts changed with blue light damage, especially of the proteins with sizes of 70 kDa and 38 kDa, marked with asterisks.

Blue light induced lipid peroxidation was assessed by immunohistochemistry detection of malondialdehyde (MDA) and 4-hydroxy-nonenal (4-HNE). These are reactive intermediates in the formation of advanced lipoxidation endproducts (ALEs). Thus, they are frequently measured as indicators of lipid peroxidation and oxidative stress. We detected an increase of MDA, 4-HNE and their adducts in the outer segment of retinas that were exposed to blue light irradiation for 12 h compared to their time-matched controls (Figure 6). A MDA adduct (ca. 75 kDa) in the irradiated sample of the OS segment fraction was increased, but decreased in the pellet (Figure 7). Other proteins (ca. 70 kDa and 38 kDa) were also influenced by blue light in OS and in the cytosolic fraction (pellet), respectively (Figure 7).

**Figure 8:**
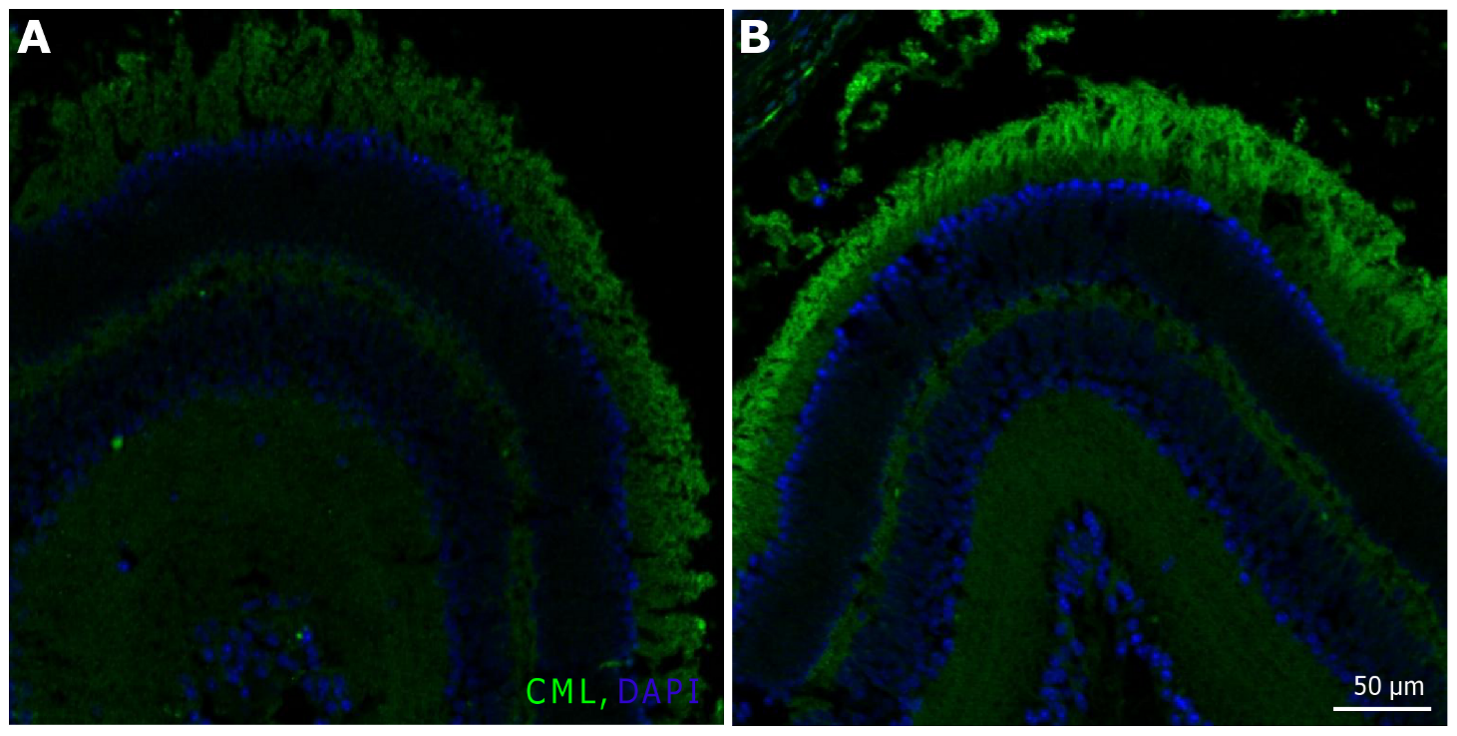
Expression of N(6)-Carboxymethyllysine (CML) was increased in OS after 12 h of blue light irradiation. **A,** Retina paraffin section after 12 h of cultivation. **B,** Retina paraffin section after 12 h of blue light exposure. In the irradiated sample an increase of CML in the OS was detected. **A–B**, scale bar 50 µm; images are representative of n = 3 **OS**: outer segments; **IS**: inner segments; **ONL**: outer nuclear layer; **OPL**: outer plexiform layer; **INL**: inner nuclear layer; **IPL**: inner plexiform layer; **GCL**: ganglion cell layer.

N(6)-Carboxymethyllysine (CML) is an advanced glycation endproduct (AGE). Under oxidative stress, AGE formation can be increased beyond normal levels. CML is the most used marker for AGEs. We detected an increased CML expression in the outer segment layer of retinas after 12 h of blue light irradiation compared to time-matched controls (Figure 8).

**Figure 9:**
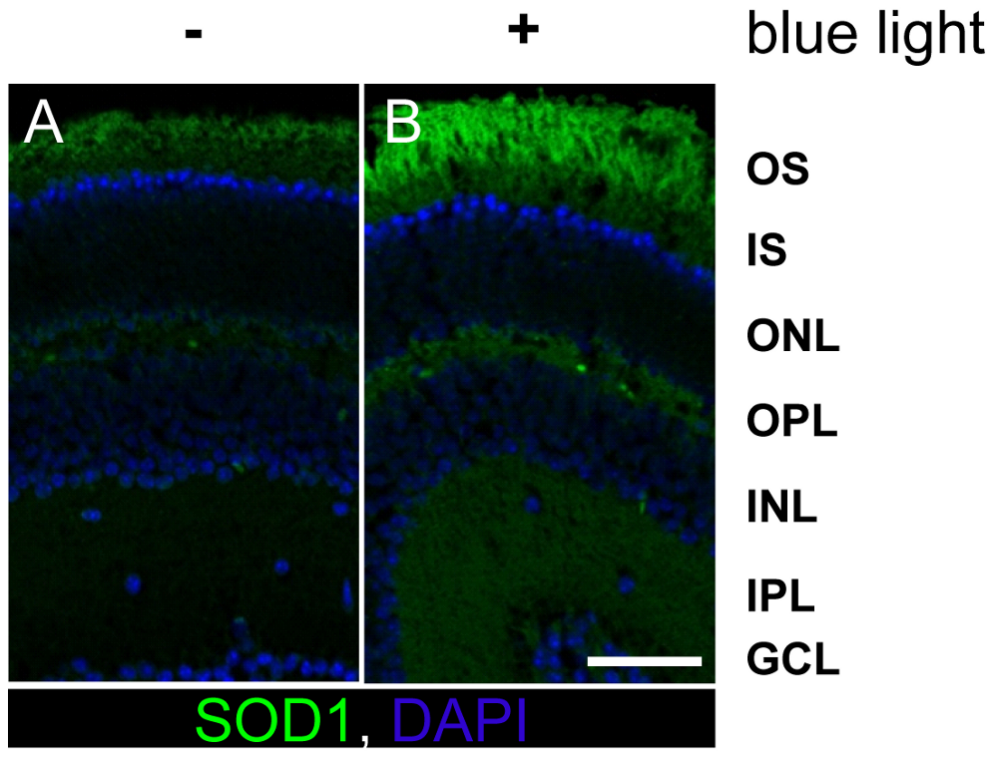
Expression of SOD-1 was increased in OS after 12 h of blue light. **A,** Retina paraffin section after 12 h of cultivation. **B,** Retina paraffin section after 12 h of blue light exposure. Under both conditions the expression of SOD-1 was higher in OS than in IS. The irradiated sample showed an increase of the protein in the OS. **A–B**, scale bar 50 µm; images are representative of n = 3 experiments **OS**: outer segments; **IS**: inner segments; **ONL**: outer nuclear layer; **OPL**: outer plexiform layer; **INL**: inner nuclear layer; **IPL**: inner plexiform layer; **GCL**: ganglion cell layer.

Superoxide dismutase 1 (SOD-1) is an enzyme that catalyzes the dismutation of superoxide anions to oxygen and hydrogen peroxide. After 12 h of blue light exposure to the retina sample, we detected that SOD-1 expression rose especially in the outer segments of the retina via immunohistochemical staining (Figure 9).

Western Blot analysis confirmed these data by an increase of the SOD-1 enzyme in the outer segment fraction compared to the time-matched control (data not shown).

### Outer segments showed a mitochondria-like membrane potential

After probing the blue light induced ROS production and observing the generation of secondary oxidized metabolites or enzymes involved in radical metabolism we wanted to test the hypotheses of mitochondria-like activity of the outer segments in dark environment and with blue light impact.

**Figure 10:**
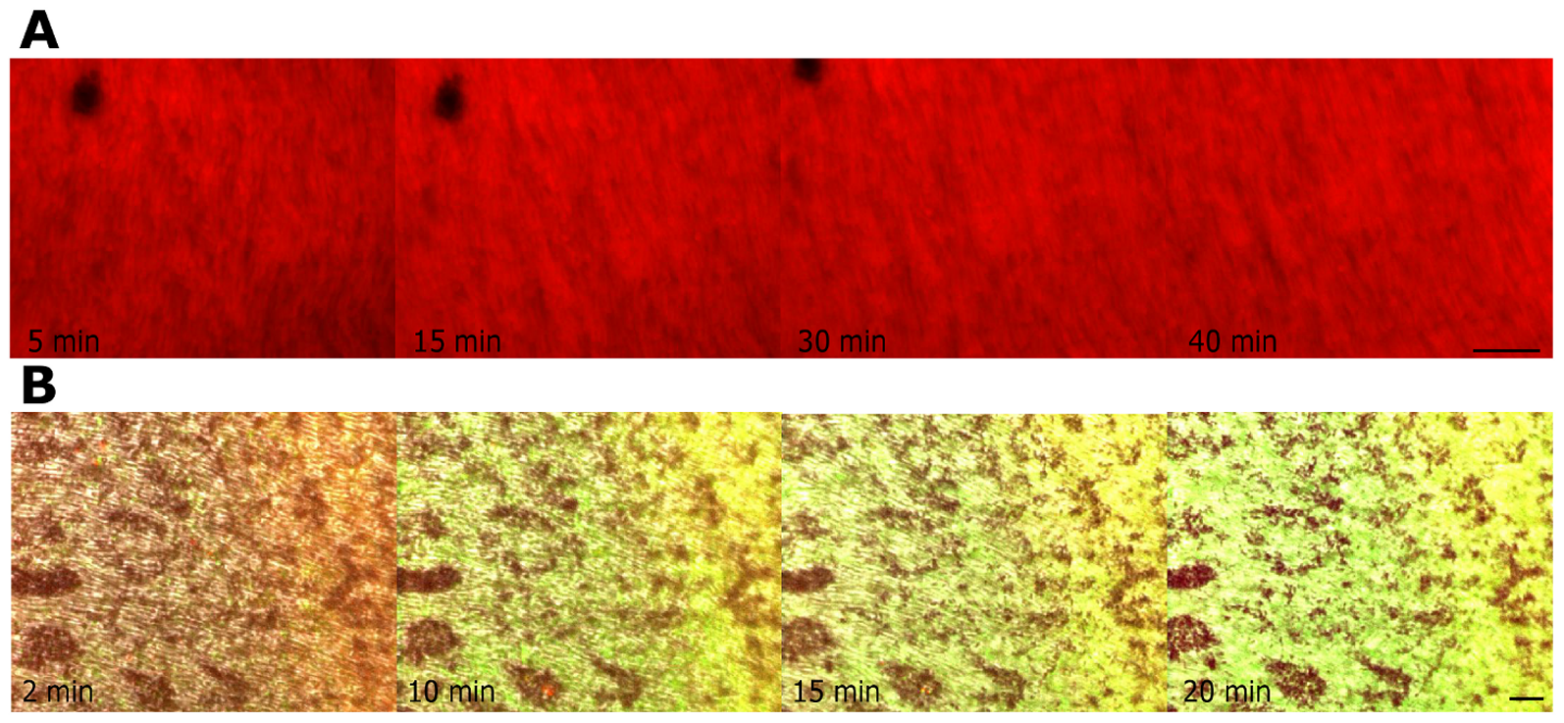
Short-term cultivation with Mitochondrial membrane potential (MMP)-dyes TMRE and JC-1. Images of RPE-OS-layer of whole mounts directly after preparation of the retina during live staining with TMRE (**A**) and JC-1 (**B**), respectively. The color of JC-1 slightly changes from orange-yellow (t = 2 min) to yellow (t = 10 min) and then to yellow-green (t = 20 min) in the pictures of merged fluorescence channels and DIC-images, which could indicate a change of a extra-mitochondrial membrane (**B**). TMRE does not show such distinctive changes in intensity after short-term cultivation (**A**). **A–B**, images are representative of three experiments; scale bar 20 µm.

To a possible intact membrane potential in the outer segment disks, we had to prepare the photoreceptors very quickly (as we know from mitochondria, where every time delay leads to depolarization). Thus, retinas were freshly prepared from animals after decapitation and enucleation. They were shortly incubated with either 10 µg/ml JC-1 or 20 nM TMRE, two markers of the MMP in living cells which also apparently stained the outer membranes of the photoreceptors. Slight changes in the color of JC-1 (from orange – yellow to yellow and then to green) were detectable which could indicate a change of the extra-mitochondrial membrane potential (Figure 10). TMRE does not show such distinctive changes in intensity after short-term cultivation (Figure 10).

**Figure 11:**
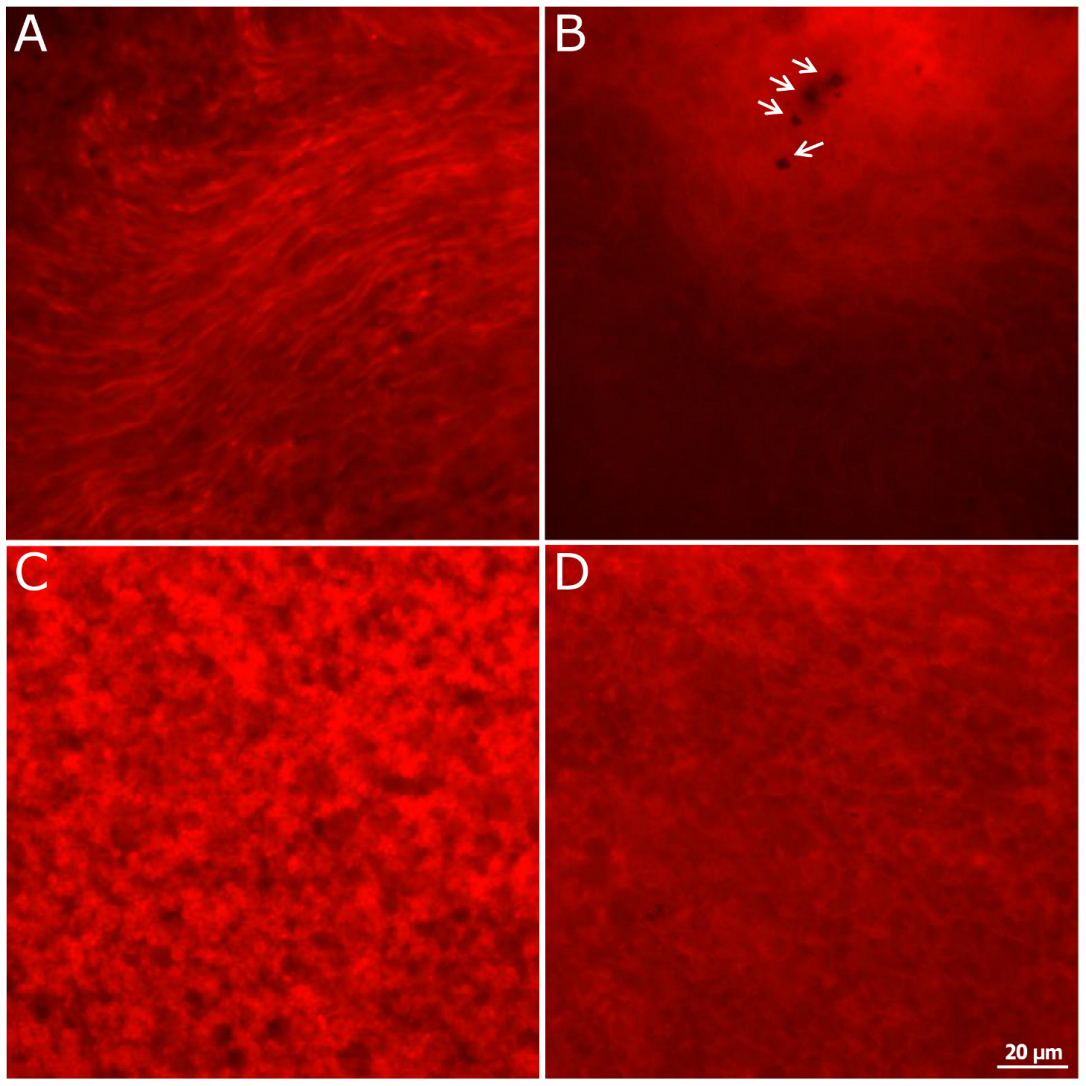
Decrease of TMRE products after 6 h and 12 h of blue light irradiation in OS. Images of the outer segment layer of whole mounts stained with 20 nM TMRE after 6 h (**A**, **B**) and 12 h cultivation (**C**, **D**). The blue light irradiated samples (**B**, **D**) showed a distinct decrease of TMRE products in the outer segment layer compared to the time-matched controls (**A**, **C**). The images are representative of 3 experiments. The arrows point to leftovers of the RPE.

**Figure 12:**
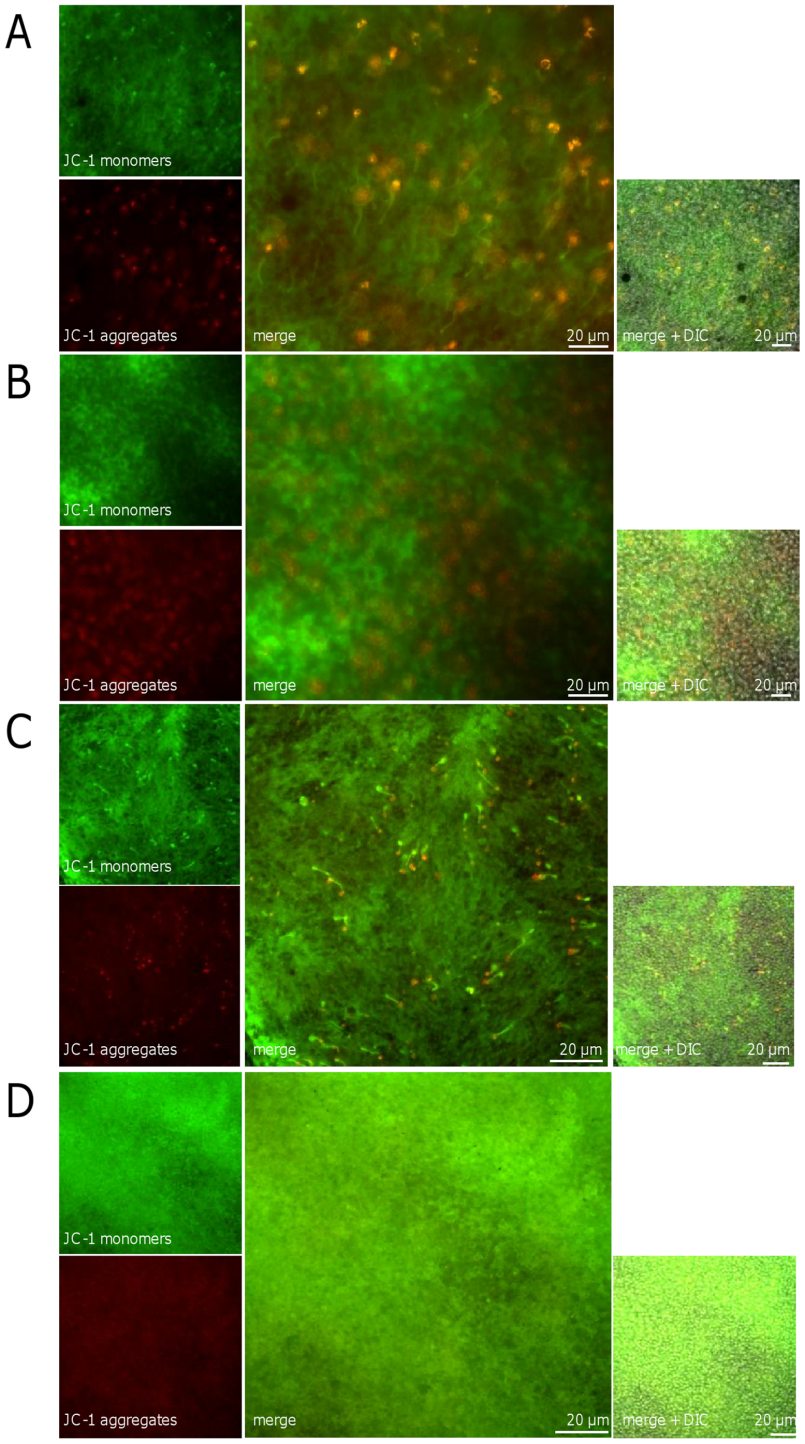
Shift of JC-1 staining after blue light irradiation in OS. Images of the outer segment layer of whole mounts stained with 10 µg/ml JC-1 after cultivation. The eyes were irradiated with blue light for 6 h (**B**) and 12 h (**D**), the time-matched controls cultivated for 6 h (**A**) and 12 h (**C**), respectively. An increase of green JC-1 monomers (sign for MMP collapse) was detected in both irradiated retinas to a greater extent than in the controls. Some fluorescent red J-aggregates (seen in healthy cells) were still visible after 12 h irradiation (**D**). In the controls the aggregates were observed as spots additionally to the staining of the outer segments in the whole mount (**B**, **D**). Images are representative of three experiments.

A longer irradiation with blue light for 6 h and 12 h showed a decrease in TMRE products after 6 h: it was higher in the irradiated retinas than in the time-matched controls (Figure 11). The green monomeric form of JC-1 (sign for MMP collapse) was present to a greater extent in both irradiated retinas than in the controls (Figure 12). Fluorescent red J-aggregates (the appearance which is seen in healthy cells) were still seen after 12 h irradiation and in the controls (Figure 12).

## Discussion

While it is a well known fact that blue light can elicit ROS generation in the retina, it is not clear exactly how and where ROS are generated inside the photoreceptors [25], [26]. ROS are diffusible and short-lived molecules. Thus, localizing the ROS signal at the specific subcellular compartment is essential for activating redox signalling events after receptor activation. ROS are involved in physiological signalling reactions, but it also accepted that excessive amounts of radicals are able to initiate vicious cycles within the cell metabolism [27], [28], [29]. This is especially true for the photoreceptors because they harbour within their outer segments a vast amount of photosensitive pigments (generating e.g. A2-PE hydrolyzed to A2E as major component of lipofuscin) [30]. In a study on isolated frog rods, Demontis et al. showed that rhodopsin in the outer segment, when activated by blue light, can produce oxidative radicals which can also lead to lipid peroxidation [31]. On the other hand a robust amount of reactive oxygen species is produced in the ellipsoid when cultured photoreceptor cells (without a true outer segment) are exposed to blue light [32].

The exact intracellular localization and quantitative relation of ROS production, however, has not been shown until now. In this paper we have demonstrated for the first time that not only the inner segment of the photoreceptors but also the outer segments directly are a source of radicals that mediate blue light-induced detrimental effects on cells which may lead to cytotoxicity. The Nox proteins are essential sources of ROS production in photoreceptor outer segments, although obviously multiple factors contribute to it. This observation was supported by experiments with the Nox inhibitor apocynin. In addition, we could show a blue light stimulated increase of the antioxidant defence enzyme superoxide dismutase 1 (SOD-1) in the outer segments of the photoreceptors.

The increased levels of MDA, 4-HNE which we also found after blue light indicate lipid peroxidation and formation of advanced glycation endproducts. Both processes indicate secondary reactions to radicals [33], [34]. Regarding structural damage in a recent paper we showed that the membranes of mouse photoreceptor outer segments get distorted after blue light irradiation [7]. This feature occurred after a longer irradiation time compared to the time scales used in the present study. Possibly, the distortion and membrane ruptures we found were caused by a longer lasting ROS attack on the PUFA containing membranes.

On the other hand it is a relatively new and hitherto largely ignored finding that the outer segments themselves contain the mitochondrial machinery for the oxidative phosphorylation [16]. Interestingly, we could corroborate the mitochondria-like staining behaviour with the mitochondrial membrane dye JC-1 which Bianchini et al. used for depicting specifically the photoreceptor outer segments (in a green colour – indicative for depolarized mitochondrial membranes) [35]. In the present paper we could add the functional aspect to this morphological information: the outer segments are not only specifically depicted by JC-1 but also react functionally like mitochondria stained with this dye - they lose their red colour (indicative for polarized membranes of well functioning mitochondria) over time (due to anoxia in the explanted whole mount) and get yellowish and later showed the full green colour of depolarized membranes. This was corroborated by a further indication of membrane depolarization via another mitochondrial membrane dye we used, TMRE. At this time one can only speculate whether this unique staining behaviour of the outer segment membranes is due to a proton electrochemical potential difference across the disk membrane which was already shown by Uhl and Desel in 1989 [36]. It now accepted that many types of rhodopsins are capable to pump protons although with low efficiency [37].

In the light of the present results, the process of constant disk renewal should therefore be a major function of the inner segment mitochondria because shedding of outer segment membrane disks is prone to interference by blue light and ROS and requires a vast amount of energy: the photoreceptors as a whole consume 3–4 times more energy than all other retinal or central nervous system cells [38], [39]. However, the present results indicate that not only the mitochondria of the inner segment but also the outer segments themselves should be responsible for this very high oxygen consumption of the outer retina [8] and for a high ROS production in addition to Nox proteins. The high levels of oxygen coming from the normally well-perfused choroidea might promote oxidative stress. In addition to this, we have found that oxidation enzymes in outer segments (able to produce ROS) like Nox-2 increased after blue light impact. Members of the Nox family of enzymes generate superoxide radicals by one electron-reduction of molecular oxygen by NADPH [40]. Bhatt et al. (2010) also found that in the photoreceptors Nox-4 increased most among the Nox family after stressing mouse retina explants with serum deprivation. The authors found that both rods and cones reacted: however, they did not differentiate outer and inner segment of the photoreceptors [12]. Also Usui et al (2009) could show in transgenic mice that Nox plays a central role in cone cell death in retinitis pigmentosa – an effect which they could reduce by an inhibitor of Nox [13].

Blue light rapidly induced ROS formation in retinal explants after 0.5–1 h. This is most probably due to increased NADPH oxidase activity. The classical NADPH oxidase activation in macrophages involves translocation of phosphorylated cytosolic subunits to the membrane thus forming an active Nox-2 complex [41]. A similar process might be responsible for the rapid increase of ROS formation in our system. The increase in Nox-2 and Nox-4 mRNA and protein expression beginning after 1 h might represent a cellular adaptation to prolonged blue light exposure. Increasing amounts of Nox proteins might be needed to form additional NADPH oxidase complexes. Part of the observed Nox-2 expression might also represent monocytes/macrophages. Furthermore, increasing evidence support a role of Nox-4 in mitochondrial ROS release of different cell types [42], [43]. Therefore, Nox-4 might contribute to mitochondrial release of ROS in our retinal explant model as well.

Our experiments show that blue light possibly induces ROS in outer segments via NADPH oxidase as well as the mitochondria-like activity of the outer segments. The cross talk between NADPH oxidases and mitochondria-like activity may stimulate NADPH oxidases. An example of such a cross-talk between NADPH oxidases and mitochondria has been recently shown with SOD-2 depletion causing an increase in NADPH oxidase activity, whereas SOD-2 over-expression reduces activation of NADPH oxidases and NADPH-generated ROS [44]. SOD-1 deficiency leads to aging in tissue with changes attributable to an elevation of ROS, also seen in the retina of *Sod1−/−* mouse retina [45].

In our further investigations we will concentrate on the differentiation between ROS sources Nox and respiratory chain in photoreceptor outer segments. The exact nature of the mitochondria-like appearance of the outer segments and their extra-mitochondrial aerobic metabolism will also be examined in these studies.
